# Supplementary material for: Are carbon emissions trading and green financial instruments synergistic? -Comprehensive quantitative research based on content analysis
Source: PLoS One. 2024 Mar 7;19(3):e0298601. doi: 10.1371/journal.pone.0298601 (PMC10919601; doi:10.1371/journal.pone.0298601)
Supplement: S1 Data — (ZIP) [file pone.0298601.s001.zip › Data/地级市Z_F工作报告.zip!/║ú─╧/╚2╤╟╩╨/└┤╫╘╣1⁄2╓┌║┼í╛┬φ┐╦╩2╛▌═°í┐_2015.docx]

各位代表：

现在，我代表市政府向大会报告工作，请予审议，并请市政协委员和其他列席人员提出意见。

2014年工作回顾

过去的一年，面对复杂多变的经济形势，市政府在省委、省政府和市委的正确领导下，在市人大、市政协的支持监督下，与全市人民一道，锐意进取，积极作为，取得了新的发展成绩。全市实现生产总值404.4亿元，按可比价格计算，增长5.5%；其中第一、二、三产业增加值分别增长5.2％、14.1％和3.1％，三次产业结构为13.5:20.3:66.2。全市地方一般公共预算收入77.8亿元，增长15.2%。一年来，我们主要做了以下工作：

一、全力推进项目建设，确保投资持续增长。坚持实施“六个一”责任机制，全市重点项目超额完成年度投资计划，带动全市固定资产投资完成629.6亿元，增长20.3%。其中，基础设施投资78.3亿元，增长32.1%，商贸金融、旅游业投资分别增长97.7%和21.1%。海棠湾国际购物中心、三亚红树林度假会展酒店等项目已建成，南航三亚总部基地综合楼建设加快推进，海棠湾亚特兰蒂斯项目已开工，梦幻不夜城等项目前期工作有序推进，三亚现代有轨电车项目线网规划已获批准。

二、加快传统产业升级，夯实产业经济基础。旅游业发展质量进一步提升，全年接待过夜游客1352.8万人次，增长10.2%，全市旅游总收入269.7亿元，增长15.6%。海洋旅游、免税购物、婚庆蜜月、医疗健康、文化会展等产业加快发展。房地产业结构调整继续推进，经营性地产在商品房网签销售面积中所占比例从2013年的18.7%提升到21.8%。商贸产业进一步发展，全市免税品营业收入达到33.7亿元，增长28%；社会消费品零售总额141.5亿元，增长14.6%。全市大型商业网点面积增加19.4万平方米。月川、港华农贸市场已完成升级改造，吉阳农产品综合批发市场即将建成投用。严格落实支农惠农强农政策，全年累计发放涉农补贴1.2亿元，增长10%。全市农民专业合作社组织达到421家，“三亚芒果”获批国家地理标志证明商标。积极推进崖州中心渔港项目建设，大力开展深海远海渔场捕捞，海洋产业发展逐步提速。

三、积极培育新兴产业，做大做强园区经济。全年实现金融业增加值44.3亿元，增长18%，金融业税收收入增长73.2%。中信银行三亚分行已开业，7家小额贷款公司获批筹建，3个保险分支机构进驻三亚。出台了专业化特色产业园优惠政策。在高新技术产业园设立了上海股权托管交易中心三亚企业挂牌孵化基地，已有8家企业挂牌。现代服务业产业园、创意产业园、高新技术产业园等园区基础设施和配套服务进一步完善，吸引赛伯乐智慧通信科技园、中国电子海棠湾信息安全产业园、瑞士洛桑酒店管理学院、德国医疗公园、国家杂交水稻三亚南繁综合实验基地等重要项目落户，三亚百泰纳米生物科技研发基地实现投产，中兴通讯、用友软件稳步发展，省动漫产业基地新引进19家企业，“三亚云港”已经启动。

四、加强城乡规划建设，提高城镇化发展质量。出台《关于进一步加强规划管理集约利用土地促进城乡统筹科学发展的意见》，规范土地规划、开发、利用机制。完成了原各区镇总体规划、镇区控规以及所有村庄建设规划的编制工作，控规覆盖率达到95%以上。城乡基础设施建设力度加大，绕城高速公路槟榔连接线实现通车，技工学校至华盛水泥厂道路改造、迎宾路、机场路拓宽改造、春光路改造、解放路改造地上部分等工程已完工，西环高铁三亚段、南山港滚装码头、凤凰岛国际邮轮港二期工程、红沙隧道公路、三环路凤凰机场段等项目有序推进。加快城乡公交一体化发展，行政村通公交车率达到79%。全市新增雨污水管道48公里。城乡供水等项目顺利推进。大隆灌区配套工程、中小河流整治工程、田洋灌排渠道整治工程等水利项目加快实施。天涯力村全国农村集体土地产权制度改革试点项目安置区建设完成，海棠湾风情小镇、育才特色风情小镇建设和鹿回头社区、榕根社区改造稳步推进，吉阳六盘村“美丽乡村”启动建设。

五、进一步强化城市管理，着力提升城市形象。围绕让路于民的目标，大力开展交通综合整治，查处各类违规行为。开工新建各类停车场25个，新增停车位5000多个。智能交通系统指挥平台建设有序推进。加大了公交车更新力度，实施公交候车亭和站牌维护改造。继续强化违法建筑管控，全年组织拆除违法建筑1400多栋、63万平方米。加强旅游服务市场监管，严厉打击“零负团费”、强迫消费、商业贿赂等违法违规经营行为，强化海上联合执法，突出海鲜排档、水果摊点、旅游购物点等重点场所的监管。出台《三亚市旅游市场违法违规经营行为举报奖励办法》，强化社会监督，推进旅游经营主体诚信建设。坚持让绿于民、让景于民、让海于民，加强对三亚湾区域和市内主要广场的整治、保洁和绿化，新建、恢复和改造城市绿地62.6万平方米，对占用人行道、沙滩、绿地、河道乱搭乱建的各类设施和户外广告进行了清理，市容市貌得到改善。

六、加大生态环保力度，积极建设美丽三亚。生活垃圾焚烧发电一期项目、生活垃圾填埋场渗沥液处理站改扩建项目已投入试运行，餐厨垃圾处理项目即将投入试运行。吉阳垃圾转运站已建成，全市已有5座垃圾转运站投入使用，市―区―村三级管理模式下的垃圾收集、转运、处理体系基本形成。进一步加强了饮用水水源地环境监测和保护，加强了空气质量监测和大气污染防治。认真开展固定资产节能评估和审查登记，对重点用能单位、行业主管部门开展节能目标责任考核，加强了重点污染源防治和在线监控。三亚市自然资源资产负债表编制工作稳步推进。大力实施“绿化宝岛”行动，全年完成造林任务8300多亩；推动“退果还林”工程，完成造林种草任务1万余亩；强化生态公益林管护，全市生态公益林面积达到72.9万亩；开展生态补偿试点工作，山区受益群众4600多人。实施三亚湾东段人工补沙试验工程，拆除了红沙海域340家养殖渔排和海鲜排档，推进“退养还海”。

七、全面深化改革开放，增强发展内生动力。有序实施行政区划调整，四个新设区已挂牌成立。按照精简高效、重心下移、权责一致的原则，科学配置区级机构职能，初步下放给区一级行政审批事项54大项、117小项和管理服务事项94小项，并从市级划转200名行政编制和人员到新设各区。按照新一轮政府机构改革的要求，认真制定市政府职能转变和机构改革方案。事业单位分类改革稳步推进。开展工商注册制度便利化改革，新登记各类市场主体增长56.8%。继续推进服务业综合改革试点，设立现代服务业产业园。建立三亚农户征信系统，继续开展农民小额贷款发放和贴息工作。优化国有经济结构布局，市公交集团、水务集团组建工作基本完成，成立了市信息化基础设施投资建设发展有限公司。积极推进优质企业参与市属国企改制重组，发展混合所有制经济。对外开放取得新成效，获批试行扩大游艇开放水域，航油保税政策落地实施。博鳌亚洲论坛年会期间的重大国事活动、2014世界旅游旅行大会、第五届三亚国际数学论坛、第三届三亚国际财经论坛等活动的成功举办，进一步提升了三亚国际化形象。

八、切实保障和改善民生，统筹发展社会事业。全年市级财政预算安排民生支出75.9亿元，增长20.7%，占地方一般公共预算支出的72.4%。与此同时，三公经费支出下降了35.2%。全市新开工建设保障性住房6357套，竣工交用1801套，完成农村危房改造1800户。全年新增城镇就业岗位3.3万个，农村富余劳动力实现转移就业1.5万人，城镇登记失业率为1.48%。多措并举提升居民收入，全年城镇常住居民人均可支配收入26860元，增长8.2%；农村常住居民人均可支配收入11285元，增长11.9%。全年居民消费价格涨幅2.9%，物价水平保持基本稳定。继续扩大社保覆盖面，城乡居民医疗保险参保率达到99%以上；建立了城乡一体的基本养老保险制度，全市城乡居民养老保险参保率达到99.3%，被征地农民养老保险实现应保尽保；城乡居民低保标准进一步提高。加强中小学校基础设施建设，第十小学、特殊教育学校等挂牌成立，完成第四中学整体搬迁，全市新增高中学位230个；区属幼儿园建设进度加快，育才中心幼儿园建成开园。市人民医院内科楼、农垦三亚医院外科综合楼建成投用，三亚国际友好中医疗养院基本建成，市妇幼保健院整体搬迁工作进展顺利，解放军四二五医院整体改建加快推进；巩固了公立医院对口支援基层卫生院的长期合作帮扶机制；全面完成省下达的人口计生工作任务；三亚被评为国家慢性病综合防控示范区。加快实施文化惠民工程，着力开展全民健身活动，公共文化服务水平得到提升。农村饮水安全、改水改厕、扶贫攻坚、沼气工程等工作取得新进展。食品药品安全监管进一步加强。严厉打击毒品犯罪、“两抢一盗”犯罪，开展“铁拳禁赌”专项行动；启动社会治安新型防控模式，提升突发案件的快速处置能力。认真做好法律援助工作，进一步畅通信访渠道，及时排查调处各种矛盾纠纷，社会保持和谐稳定。妇女、儿童、青少年、老龄、残疾人、红十字、志愿者事业有了新发展，民族、宗教、侨务、口岸、气象、地震、人防、档案、史志、文物保护等工作取得新成绩。

九、加强政府作风建设，努力提升行政效能。自觉接受人大、政协和社会各界监督，一年来办复市人大代表建议80件、市政协委员提案144件，办复率100%。继续开展市级行政审批事项清理，减幅达到58.6%。整合优化行政审批流程，平均办理时间由3.2个工作日缩短至2.6个工作日，52.6%的审批事项实现全流程网上审批。建立建设项目并联审批机制，审批时限由900多天减少到200天左右。居（村）委会便民服务站实现全覆盖。修订市政府工作规则，进一步完善市政府决策程序。强化工作督查督办，严格执行问责制度，认真开展绩效考评，行政效能得到提升。认真开展群众路线教育实践活动，大力整治“四风”，解决了一批涉及群众切身利益的实际问题。全面推行预算项目库改革，公开了市本级财政预决算、部门预决算以及三公经费预决算信息，强化了财政投资评审和预算绩效评价。进一步加强政府廉政建设，加大了审计、监察工作力度。

各位代表，在过去的一年里，我们努力克服多重困难，取得了来之不易的成绩，这是省委、省政府和市委正确领导的结果，是市人大、市政协和社会各界大力支持的结果，是全市上下凝心聚力、真抓实干的结果。在此，我谨代表市政府，向全市各族人民，向人大代表、政协委员、各民主党派、人民团体和社会各界人士，向中央和省驻三亚各单位，向驻市部队、武警官兵、公安政法干警，向所有关心支持三亚发展的国内外朋友，表示衷心的感谢，并致以崇高的敬意！

在看到成绩的同时，我们也必须正视发展中存在的困难和问题。一是经济总量偏小，发展质量不够高，产业结构性矛盾突出，抗风险能力较弱。二是基础设施建设滞后，特别是交通设施不能满足城市发展需求。三是城市管理和服务水平亟待提升。四是征地拆迁难、违法建筑管控难等问题仍未得到根本解决。五是生态环保压力增大，尤其是污水、垃圾处理和山体、森林、河岸、海岸管控与保护能力急需增强。对于这些问题，我们将高度重视，并采取有力措施，切实加快解决，让人民群众见到实实在在的成效。

2015年主要任务

2015年是“十二五”规划收官之年，也是三亚推进发展提质增效的重要一年。中央和省经济工作会议指出，要坚持以提高经济发展质量和效益为中心，主动适应经济发展新常态，把转方式调结构放到更加重要的位置。从三亚实际来看，随着城市规模和人口、经济总量的不断扩大，简单粗放的发展方式注定将难以为继。为了更好地将宝贵的自然条件转化为发展优势，我们必须更加科学、精细、长远地谋划城市发展。当前，市委按照省委罗书记的指示精神，结合三亚实际，提出了建设国际化热带滨海旅游精品城市的新目标，突出强调“精品”二字，对城市发展提出了更高要求。市政府要围绕这一目标，千方百计抓好落实。今年政府工作的总体要求是：全面贯彻落实党的十八大、十八届三中、四中全会和习近平总书记系列重要讲话精神，努力争创中国特色社会主义实践范例，按照市委的决策部署，坚持生态立市、产业强市、依法治市、文化兴市，以提质增效为重点，以改革创新为动力，加快转方式调结构，推进新型城镇化发展，强化城市治理管理，增进人民福祉，努力建设国际化热带滨海旅游精品城市。今年我市经济社会发展的主要预期目标是：生产总值增长8%；地方一般公共预算收入增长11%；固定资产投资增长12%；全市接待过夜游客增长10%；社会消费品零售总额增长13%；城镇常住居民人均可支配收入增长8.5%，农村常住居民人均可支配收入增长10%；居民消费价格涨幅控制在4%以内；完成“十二五”节能减排控制目标。为实现上述目标，要重点抓好以下几个方面的工作：

一、坚持推行绿色发展，加强生态文明建设。市委、市政府将出台“关于进一步加强生态环境保护工作的决定”，政府相关职能部门要据此制定分管领域的具体行动计划并加快实施，逐步形成绿色低碳循环发展新方式。

（一）严格落实节能减排任务。加强大气污染监测与防治，严格控制建筑施工和道路扬尘，落实“黄标车”、无标车限行和淘汰制度。加快推动崖城、红塘湾等水质净化厂和主城区雨污水收集排放系统工程、三亚湾新城水质净化厂配套管网工程建设，着力提高污水收集处理率。大力强化污水排放管理，严厉查处偷排、乱排和超标排放行为，争取年内全面完成污水排河排海整治工作。加快推进农村垃圾收集全覆盖工程，继续建设和发挥好生活垃圾焚烧发电以及餐厨垃圾处理项目的作用，争取全市的生活垃圾和餐厨垃圾得到100%收集处理。加快推进建筑垃圾处理项目的前期工作。落实节能目标责任制，抓好重点领域节能，有序推进重点用能单位的能耗动态监测项目建设，制定能耗限额标准。

（二）加大生态环境保护和恢复力度。继续实施“绿化宝岛”行动，推动“退果还林”工作，确保完成8000亩退果还林任务。大力推广生态果林种植模式。继续开展森林生态效益财政补贴试点工作，扩大生态补偿范围。进一步加强大隆、赤田等饮用水水源地环境保护。积极推进海棠湾内河水系工程建设，加快安排开展全市各河流水系的治理。做好红树林生态恢复工作。加强海洋生态环境特别是珊瑚礁生态系统和三亚湾等海湾、海岸带保护，积极探索海洋生态资源利用补偿机制，争创国家海洋生态文明示范区。

二、加快产业转型升级，打造新经济增长点。通过传统产业提质和新兴产业增量来发掘和培育新的增长点，构建适度多元的特色产业体系。

（一）提升传统产业发展质量。以海棠湾国际购物中心和正在建设中的亚特兰蒂斯项目为典范，积极打造旅游精品项目；大力发展海洋旅游、医疗健康等产品，力求在邮轮游艇、低空旅游等方面取得新突破；进一步做好旅游市场宣传促销，深入开发国际市场，精确定位目标客源地，创新旅游营销方式，有效构建起4小时、8小时、12小时旅游圈，提升三亚国际市场竞争力。着力推进房地产业转型升级，大幅减少商品房类住宅用地供应，适度减少酒店类用地供应，优化土地供应结构；提高企业进入门槛，注重培育商业、金融、智慧、商务办公、文化教育、健康养生等支撑新型产业发展的地产。加快发展热带特色现代农业，推动国家冬季瓜菜基地和常年蔬菜基地建设，推广柏盈兰花种植基地、兰德玫瑰风情产业园等花卉生产经营模式；抓住规划建设国家级南繁基地的契机，吸引大型种业企业、研发机构落户三亚，实现南繁育制种产业化；抓好农产品质量安全监管和营销推介工作，发挥农业龙头企业和示范合作社的带动作用，着力推进农业品牌建设和电子商务发展；完成崖州中心渔港搬迁工作，继续引导和扶持更新改造大吨位渔船，发展外海远洋捕捞业；继续推进海洋牧场建设，加快海钓等休闲渔业发展；结合美丽乡村建设，扶持发展休闲观光农业、循环生态农业等新业态。

（二）增强新兴产业总体实力。一是加快金融业发展。继续吸引各类金融机构进驻，有序引导民间资本进入。深化改革创新，优化金融发展环境。整合保险资源，扩大保险业对我市重点产业、社会管理、民生等方面的服务保障作用。积极培育上市资源，力争年内实现企业挂牌“新三板”。二是推动总部经济发展。完成南航三亚总部基地综合楼建设，积极推进阳光金融广场等项目，支持中铁子悦薹商务写字楼提升利用率，争取中航工业南方区域总部等一批总部企业落户三亚。三是优化产业园区发展。在对我市已有产业园区功能定位进行认真分析的基础上，对全市的产业布局进行调整。创意产业园要在现有的产业基础上，借助临港优势重点发展海洋资源开发、水产品加工、游艇制造加工及展销等产业；高新技术产业园侧重发展旅游创新创意等智慧产业；海棠湾现代服务业产业园着力打造集旅游服务、医疗健康、免税购物、总部经济、文化会展、金融服务为一体的特色现代服务业园区。加强园区配套设施建设，积极引进优质产业项目落户，加快形成我市新的产业体系。

三、着力优化投资结构，稳步扩大内需规模。把握好国家和省宏观调控政策导向，更加注重优化投资结构。突出投资对经济发展的关键作用，增强消费对经济发展的基础作用。

（一）发挥投资导向作用。坚持“以亩产效益论英雄”，提升项目质量，集约节约用地。科学编制投资和重大项目计划，大力争取中央、省项目投资和政策扶持，主动对接一批既能拉动当前经济增长，又能增强发展后劲的大项目、好项目，力争将更多的大型基础设施项目列入省和国家“十三五”盘子。根据《三亚市社会投资项目参考经济技术标准》，积极引进符合三亚发展战略的项目，特别是占地少、无污染、高附加值的产业类项目。今年安排市重点项目123个，安排年度投资计划423.7亿元。

（二）加强项目建设管理。一是抓好项目审批。不断完善建设项目并联审批机制，成立项目审批代办中心。探索建立建设项目可视化监管新模式，做到项目审批“提前介入、全程陪同、实时督办”。二是保障用地供给。加快项目用地报批进度和交付进程，加大违法建筑清理力度，提高征地拆迁工作效率。三是落实项目资金。对社会投资项目，积极搭建“政银担企”对接平台；对政府投资项目，通过多种渠道加大资金筹措力度，加快完善政府投资体制，探索推行政府投资与社会资本合作（PPP）模式。四是强化管理责任。继续坚持市级领导包点项目和“六个一”责任机制，以问题为导向，抓好项目督查工作。严格实施政府投资项目审计监管，加强项目全程质量、安全和工程监理。

（三）积极拓展消费增长点。依托海棠湾国际购物中心，进一步放大购物免税政策效应。加快推进亚龙湾奥特莱斯项目，推进亚龙湾、槟榔河、凤凰水城的商业街建设。积极推进三亚罗牛山农产品加工物流产业园、崖州瓜果菜交易中心等一批重点物流项目建设，结合旧城改造继续推进第一、第三等农贸市场升级改造，完善社区商业网点布局。积极发展文化、信息、医疗、康体、养老等服务性消费，培育新的消费热点。

四、加快城市建设步伐，推进新型城镇化发展。在科学规划的指导下，大力加强城市建设，加快实施片区改造。

（一）坚持科学规划引领。积极适应撤镇设区等方面的变化调整，进一步加强规划编制、审批和批后管理工作。一是要积极推进城市总体规划修编的前期工作。二是要加快落实各类城市功能布局规划，如商业网点、公交场站等。同时，要通过建筑、景观、公共空间等方面的高水平城市设计来详细指导精品城市建设。三是要切实落实村庄规划。四是要精心编制“十三五”规划。全面评估“十二五”规划实施情况，研究编制“十三五”规划纲要和14项“十三五”行业或专项规划。要按照“多规合一”的思路，切实加强经济社会发展规划、城乡规划、土地利用规划以及基础设施规划、产业发展规划、生态环保规划之间的统筹协调。

（二）大力完善基础设施建设。加快撤镇设区后的整体城镇化步伐，推动基础设施城乡一体化建设。加快推进红沙隧道公路、龙岭路、三环路、芒果路、胜利路、绕城高速公路凤凰机场连接线、榕根桥、月明桥、跃进桥等工程，积极推动海榆东线、海榆西线三亚段和凤凰路南段、金鸡岭路延伸段改造工程，推动海上巴士交通系统建设，抓紧研究开展凤凰路与迎宾路的交叉下穿立交、鹿回头半岛与三亚湾的下穿隧道、亚龙湾第二通道等项目前期工作，进一步优化交通路网体系。加快推进凤凰机场改扩建和西环高铁三亚段建设，尽早启动机场、动车站综合交通枢纽项目，抓紧开展三亚旅游观光铁路、现代有轨电车等项目的前期工作。加快西部供水二期工程和青田水厂供水二期扩建工程进度，继续推进电力、燃气等设施建设。加快“两网一中心”等信息化基础设施建设，重点提升三亚政务服务、城市管理、旅游公共服务以及景区景点、宾馆酒店等方面的信息化水平，把信息化作为新型城镇化的重要支撑来抓。

（三）加强城市绿化美化工作。坚持让绿于民、让景于民、让海于民。做好三亚湾等区域原生植被的恢复和保护工作，完成迎宾路、凤凰路、榆亚路等城市主干道绿化提升的规划设计并分步实施。加强公共绿地管护，严格执行“绿线”管理制度，对全市各类绿地划定范围控制线，禁止擅自占用或改变其用地性质。加快推动鹿回头广场、大东海广场和白鹭公园等广场、公园的升级改造，完善其景观、照明和休闲服务设施。策划开展三亚湾、三亚河、临春河等“一湾两河”沿岸景观美化和灯光亮化提升工作，进一步完善沿岸休闲服务设施。

（四）加快推进棚户区改造。重点推进港务局码头及其周边渔港、水居巷、鹿回头社区、月川社区、东岸村、海坡村、临春村、藤海村、洪李村等棚户区改造，并借此建设一批宜居宜商的风情小镇。加快育才省级特色风情小镇示范镇建设，完善其道路交通、饮水安全等设施。积极推进海棠区青田、林旺南、林旺北、龙海、龙江等小镇的建设和搬迁工作，将推进搬迁入住作为今年改造工作重中之重的任务来抓，引导扶持居民在民宿客栈、手工艺制作、商业零售、特色餐饮等方面有所发展，着力构建管理有序、服务完善、生活便利、社会和谐的新型现代社区。启动城边村改造参与企业退出机制，提升资源配置效率。以吉阳六盘村等村庄为重点，加快“美丽乡村”建设。

五、加强城市治理管理，打造旅游精品城市。深入开展“城市治理管理年”活动，努力解决各类突出矛盾和顽疾问题。

（一）全力打好五大关键战役。一是坚决打好交通环境治理管理战役。继续加快停车设施建设，力争其服务范围能够合理地覆盖城市的每一个区域。在城市中心区域鼓励实行科学的停车收费管理。持续开展对乱停乱放行为的治理管理，交警和各区市容执法人员要加快建立健全相关的巡查、处罚常态化机制。着力规范电动车行车秩序，继续严格实施工程车、大货车、农用车等车辆限行措施，严厉查处闯红灯等违规行为。继续查扣和销毁非法营运的三轮车、摩托车、电动车并建立常态化管理机制。二是坚决打好旅游市场治理管理战役。继续严查严打旅游市场违法违规经营行为，大力查处景区、购物点等各类旅游消费场所涉及的商业贿赂，严防欺客宰客。集中规划建设一批管理规范、服务到位、游客喜爱的海鲜排档和水果市场。各区都要在年内完成水果摊点的归市经营，零散的水果摊点一律取缔。加快推出海鲜排档运营服务标准，坚决淘汰服务水平低、市场信誉差的经营者。积极打造以12301、12345热线为载体的综合服务平台，认真实施《三亚市旅游市场违法违规经营行为举报奖励办法》，健全举报投诉快速处理和反馈机制，引导市民游客参与监督管理。三是坚决打好涉海旅游治理管理战役。进一步完善海洋部门牵头、各部门联动的海上联合执法机制。海洋部门要科学划分旅游用海区域，文体等部门要研究制定和完善潜水等旅游项目服务标准，要按照市场化的原则实行企业准入和退出机制。继续强化对非法涉海旅游项目的整治。四是坚决打好市容市貌治理管理战役。集中清除三亚河、临春河等内河两岸、南边海等海岸带区域以及违法占用公共绿地、公共道路乱搭乱建的各类设施，大力整治占用河道和沿岸区域堆放建筑材料、建筑垃圾的行为，全面清理和整治占道经营。继续推动拆违透绿、拆围建绿工作，修订城市围墙、围栏建设标准。加强海岸沙滩、背街小巷、城中村、城乡结合部等重点区域的环境卫生整治。加强施工场地管理，严禁工程车辆带泥上路。规范管理城市沿街立面、店招店牌，继续清理户外广告。五是坚决打好违法建筑治理管理战役。坚持严查存量、严禁增量，继续开展“铁锤行动”，对违法建筑保持严查狠打态势。健全以各区政府为主，市综合执法、公安等部门配合的打击、管控违建责任机制，强化责任倒查和责任追究。今年内各区违建存量要减少一半以上。此外，各区要对辖区内的农村宅基地进行逐户登记，要切实强化农村建房报建审批管理。

（二）积极改进治理管理方式。大力推进网格化管理，把涉及到市政设施、停车、市容、环卫、违建等各个方面的城市管理内容纳入网格化体系。加快推进数字化视频监控的融合，加强数字化城管、数字食安等系统的应用，着力完善相关工作流程，提升城市管理的技术水平。结合撤镇设区工作，继续推进行政执法体制改革，明确市、区两级执法责任和权限，进一步规范执法程序。抓好执法人员教育培训和队伍建设，尤其是要在年内大幅强化公安交警等执法人员的任前教育和业务培训，努力塑造文明、专业、果敢的执法队伍形象，同时努力提升协警等人员队伍的工作水平。以中央提出赋予设区的市地方立法权为契机，加强与人大的沟通联系，提前开展有针对性的谋划研究，为逐步建立起规范化的治理管理长效机制做准备。

（三）努力建设文化三亚、文明三亚。大力挖掘天涯文化等独具特色的地域文化，加大“非遗”保护与传承力度，支持有关团队创作、推广一批优秀原创文艺精品，扶持三亚琼剧、崖州民歌、黎族苗族山歌等地方文化的传承发展，打造三亚本土文化品牌。积极推进三亚千古情和兰花世界文化旅游产业园等文化旅游项目建设，加快南海佛学院前期工作，重点打造有影响力的文化交流与体育赛事活动。完成三亚市群众艺术馆建设，加快海滨影剧院项目改造，推动文化广场、文化主题公园建设，着力完善公共文化服务体系，深入开展群众性文化活动，推行全民健身计划，丰富群众文体生活。以开展新一轮创建全国文明城市活动为抓手，以社会主义核心价值观为引领，全面提升市民文明素质和城市文明程度。积极倡导文明旅游，加大宣传教育力度，发动广大游客自觉抵制不文明旅游行为。积极营造开放包容的城市文化，增强广大市民的主人翁意识和外来者的归属感，促进和谐社会建设。

六、全力保障改善民生，建设人民幸福家园。坚持守住底线、突出重点，扎扎实实为群众办一批实事、好事。

（一）大力发展教育事业。继续推进中小学校办学条件标准化、规范化建设，完善学校布局调整，促进城乡教育均衡发展。积极引进、培养优秀教师人才，打造优秀教师队伍，全面开展与上海市长宁区和北师大的基础教育合作交流，促进我市基础教育改革发展。加快现有高中学校建设，采取联合办学的方式，创建一批优质普通高中学校。加快公办幼儿园建设，扶持民办幼儿园发展。积极支持高等院校和职业中专学校发展。

（二）提高医疗卫生服务水平。加快医疗卫生基础设施和信息化建设。积极推进全国城市公立医院综合改革试点工作。以提升基层医疗服务水平为基础，加快建立基层首诊、双向转诊制度。全面落实国家、省免费孕前优生健康检查和地中海贫血基因筛查诊断工作，提高出生人口素质。继续做好食品药品安全监管工作。

（三）做好就业服务工作。积极推进市、区、村（居）公共就业三级服务平台和就业服务信息化建设。加强城乡劳动者职业技能培训。重点围绕高校毕业生、农村富余劳动力、下岗失业人员、零就业家庭和失地农民开展就业服务和援助工作。落实各项创业扶持政策，推进高校毕业生创业孵化基地建设，探索完善小额担保贷款服务。力争新增城镇就业岗位2.5万个。

（四）完善社会保障体系。进一步稳定和扩大社保覆盖面，启动“全民参保登记计划”试点工作，抓好社保卡发放工作。深化医保支付方式改革，加快推行按病种付费为主的复合型付费方式，积极建立健全城乡居民大病保险和疾病应急救助制度。根据省里的统筹部署，落实好城乡居民医保省级统筹、居民养老与职工养老制度衔接转移等事项。进一步提高城乡低保救助标准。抓好“菜篮子”产品保供稳价工作，强化市场价格监管，进一步完善和落实物价上涨联动补贴机制。加快发展养老服务业，推进养老机构建设。推动妇女儿童、青少年、残疾人、红十字等事业取得新发展。

（五）强化住房保障工作。将住房保障的重心逐步转到外来从业人员居住和改善基层群众居住条件上来，积极发展公租房。今年全市计划完成棚户区改造9842户，新建公租房1万套，竣工各类保障性住房3526套，完成农村危房改造600户。继续做好保障性住房审核、分配、管理工作，大力查处违规行为。

（六）维护社会和谐稳定。深入开展法制宣传教育活动。进一步加强矛盾纠纷排查化解和信访积案化解工作，强化信访事项督查督办。全面落实安全生产责任制，加强重要行业和重点领域安全隐患排查，深入开展安全生产专项整治，严防重大事故，降低一般事故发生率。完善社会治安防控体系，加强社会治安综合治理。此外，要继续大力支持国防建设，抓好国防动员、民兵预备役和人防工作，广泛开展双拥共建活动。继续做好民族、宗教、侨务、气象、地震、新闻、统计、档案、史志等工作。

七、继续深化改革开放，增强三亚发展活力。通过深化改革和扩大开放，为经济社会发展提供良好的体制机制保障。

（一）继续完善行政区划调整工作。在市、区两级政府的实际工作中不断探索磨合，持续推动和完善行政审批、管理、执法权限的下放。强化、细化区级政府的属地责任，使各区政府在相关政策的制定、管理措施的选择上有更大的主动权，在推动区域经济社会发展中发挥出更加积极有效的作用。积极支持新设各区创新、改进社区服务和治理管理模式，抓好社区居（村）委会建设，发挥其基础作用。

（二）推进现代市场体系与国企改革。继续深化工商注册登记制度改革，推动登记注册、许可审批和后续监管无缝衔接。建立全市统一的公共资源交易平台，集中规范开展公共资源交易活动。探索创新公共服务供给模式，逐步扩大政府购买服务的规模。成立国企改革发展领导小组，制定出台相关改革方案，加强对国企改革工作的领导。继续分类推进关闭退出国企的资产处置与重组，整合优质资源，筹划组建集团公司，加快三亚市城市规划设计研究院等企业的混合所有制改制工作。推进国企薪酬制度改革，积极对外引进人才，实施“人才强企”战略。加强国企经营业绩考核。

（三）推进农村综合配套改革。力争完成农村土地承包经营权登记颁证工作，健全农村土地承包仲裁长效机制，依法推进经营权有序流转，推动土地集约化经营。深入开展天涯力村全国农村集体土地产权制度改革试点。大力发展普惠金融，稳妥推进农业保险、农民小额贷款发放工作，探索推进三权抵押贷款，鼓励和引导金融机构改善“三农”金融服务。加强法治农村建设，将农村管理纳入法治化、规范化轨道。

（四）积极扩大对外开放。抢抓机遇，提前谋划，在省委、省政府的整体部署下，努力发挥好三亚作为21世纪海上丝绸之路战略支点的支撑作用，提升对外开放水平。积极谋划和推动三亚新机场及临空经济区建设的相关工作，深入研究三亚国际门户机场功能定位。积极开辟新的国际航线。加快推进凤凰岛国际邮轮港二期工程，积极策划海上丝绸之路邮轮旅游航线，争取国家支持中资企业包租外籍邮轮经营南海无目的地航线，推动南海旅游服务基地建设。完善南山货运港及其配套设施，加快推动南海资源开发服务基地建设。积极争取放宽免签国家旅游团组团人数限制，争取海港口岸、空港口岸72小时过境免签政策。围绕海棠湾现代服务业产业园，努力争取国家在医疗养生产业等方面的政策支持。借助博鳌亚洲论坛等高端平台的作用，加快三亚首脑外交及休闲外交基地建设，扩大对外交流。

八、加强政府自身建设，提升政府服务水平。坚持按照“为民、务实、清廉”的总要求，加快推进政府工作作风和职能转变，提高行政效能。

（一）加快建设法治政府。坚持依法、科学、民主决策，进一步完善和落实重大决策听证、重要事项公示等制度，规范决策程序，强化决策责任。大力完善政务公开、效能监察、权力监督、举报投诉等相关制度，确保行政权力依法公正运行。坚持向人大报告工作、向政协通报工作制度，认真执行市人大及其常委会的决议、决定，自觉接受人大法律监督和政协民主监督，认真办理人大代表建议和政协提案。加强同各民主党派、工商联、人民团体和社会各界人士的联系。严格落实党风廉政建设责任制，推进惩防体系建设，保持反腐高压态势，严厉查处失职渎职、贪污腐败、徇私枉法等违纪违法行为。

（二）推动政府职能转变。着手推进市级机构改革，进一步理顺政府与市场的关系，突出市场在资源配置中的决定性作用。结合区划调整和行政审批制度改革，落实好国家和省规定审批事项的取消和下放工作。扎实推进三级政务和便民服务体系建设，着力加快政务服务新大厅和区级政务中心建设，加快政务服务信息化进程，力争实现全市审批工作一体化，力争市级审批事项全流程网上审批率达到80%以上。

（三）大力加强作风建设。巩固群众路线教育实践活动的成果，继续加强干部作风建设。坚持问政于民、问需于民、问计于民，真诚倾听群众呼声，真情关心群众疾苦，加强对群众最关注问题的调查研究，创新办法，改进工作，努力多办雪中送炭的实事、好事。大力弘扬求真务实之风，严防“四风”回潮，继续开展“庸懒散奢贪”整治，严肃处理不作为、乱作为行为。

各位代表，新的一年，新的征程。让我们紧密团结在以习近平同志为核心的党中央周围，在省委、省政府和市委的坚强领导下，解放思想，开拓创新，为全面建设国际化热带滨海旅游精品城市而努力奋斗！
